# The Role of Vitamin D in Primary Headache–from Potential Mechanism to Treatment

**DOI:** 10.3390/nu12010243

**Published:** 2020-01-17

**Authors:** Magdalena Nowaczewska, Michał Wiciński, Stanisław Osiński, Henryk Kaźmierczak

**Affiliations:** 1 Department of Pathophysiology of Hearing and Balance System, Faculty of Medicine, Collegium Medicum in Bydgoszcz, Nicolaus Copernicus University, M. Curie 9, 85-090 Bydgoszcz, Poland; 2 Department of Otolaryngology, Head and Neck Surgery, and Laryngological Oncology, Ludwik Rydygier, Collegium Medicum in Bydgoszcz Nicolaus Copernicus University, M. Curie 9, 85-090 Bydgoszcz, Poland; 3 Department of Pharmacology and Therapeutics, Faculty of Medicine, Collegium Medicum in Bydgoszcz, Nicolaus Copernicus University, M. Curie 9, 85-090 Bydgoszcz, Poland

**Keywords:** cholecalciferol, headache, migraine, tension-type headache, cluster headache, pain, vitamin D, 25-hydroxy-vitamin D

## Abstract

Some studies have suggested a link between vitamin D and headache; however, the underlying physiological mechanisms are unclear. We aimed to summarize the available evidence on the relationship between vitamin D and the various subtypes of primary headaches, including migraines and tension-type headaches. All articles concerning the association between primary headache and vitamin D published up to October 2019 were retrieved by searching clinical databases, including: EMBASE, MEDLINE, PubMed, Google scholar, and the Cochrane library. All types of studies (i.e., observational, cross-sectional, case-control, and clinical trials) were included. We identified 22 studies investigating serum vitamin D levels in association with headaches. Eight studies also evaluated the effect of vitamin D supplementation on the various headache parameters. Among them, 18 studies showed a link between serum vitamin D levels and headaches, with the strongest connection reported between serum vitamin D levels and migraine. Overall, there is not enough evidence to recommend vitamin D supplementation to all headache patients, but the current literature indicates that it may be beneficial in some patients suffering headaches, mainly migraineurs, to reduce the frequency of headaches, especially in those with vitamin D deficiency.

## 1. Introduction

Headache is a common symptom with a heterogeneous set of causes. According to the third edition of the International Classification of Headache Disorders (ICHD-3), we distinguish primary headaches (i.e., those without an underlying cause, accounting for 90% of all headaches) and secondary headaches, which are attributable to a specific etiology [1]. Primary headaches are one of the most prevalent neurological disorders, with an age of onset between 20 and 40 years old.

The most prevalent types of primary headaches are migraine and tension-type headaches (TTH). Migraines typically present with pulsating, unilateral, severe headache lasting from 4 to 72 h with accompanying nausea, phonophobia, photophobia, and sometimes, transient neurological symptoms [1,2]. Meanwhile, TTH mostly present with non-pulsating “bandlike” pressure bilaterally of the head, without other symptoms [1,2]. Another, albeit rare, primary headache disorder is the cluster headache (CH), with recurrent attacks lasting from 15 to 180 min up to eight times a day. CHs are characterized by severe unilateral pain, typically around the eye, with associated unilateral tearing, ptosis or other cranial autonomic symptoms and also restlessness and agitation [3]. Trigeminal neuralgia (TN) is also classed as a primary headache. TN is a chronic neuropathic pain disorder characterized by episodes of severe, short, electric shock-like headache in the area of trigeminal nerve [4,5]. Other very rare types of primary headache include primary cough headache, paroxysmal hemicrania and hemicrania continua, hypnic headache, short-lasting unilateral neuralgiform headache with conjunctival injection and tearing (SUNCT), primary stabbing headache, primary thunderclap headache, primary headache associated with sexual activity, primary exertional headache, and new daily persistent headache [1].

According to data derived from Global Burden of Diseases, Injuries, and Risk Factors (GBD) studies, headache became major public health concern worldwide. In 2016, almost three billion individuals were diagnosed with headache disorder: 1.89 billion with TTH and 1.04 billion with migraine. The global age-standardized prevalence was 26.1% for TTH, and 14.4% for migraine [2]. Other types of headache are not so frequent: CH affects up to 0.1% of the population [3] and TN up to 0.3% [4]. It is reported that chronic headache (which occurs ≥15 days per month) affect up to 5% of the general population [6]. Chronic forms are connected with medication overuse, bad response to therapies and lower quality of life [6].

Primary headache often coexists with mood disorders like depression, and places a considerable burden on society, primarily due to treatment cost and work absence or presenteeism [7]. Treatment of primary headache consists of abortive and prophylactic therapy: the aim of abortive therapy is to stop headaches, while the goal of prophylactic therapy is to reduce headache attack frequency and severity, or disease progression. Besides pharmacological treatment, several minerals, vitamins and medicinal herbs, including vitamin D are recommended as headache supplementary treatment [8,9,10].

### 1.1. Metabolism and Functions of Vitamin D

Vitamin D deficiency is an emerging global health problem, affecting approximately 30–80% of children and adults worldwide [11,12]. The deficiency can occur due to several factors, mostly by inadequate sun exposure. Other risk factors include old age, darkly pigmented skin, latitude, winter season, clothing, sunscreen, air pollution, smoking, homebound, obesity, malabsorption, renal or liver disease and medications (including anticonvulsants, glucocorticoids, human immunodeficiency virus medications, and antirejection [11,12,13]. Sufficient levels of circulating vitamin D are mandatory for the absorption of several minerals, particularly calcium, but also phosphorus and magnesium [14].

There are two forms of vitamin D: D2 and D3. First one is acquired from ultraviolet (UV) irradiation of the yeast sterol, ergosterol, while vitamin D3 is produced from 7-dehydrocholesterol in the skin after exposure to UV radiation [14]. Vitamin D metabolism starts in the liver where it is converted to 25(OH)D by vitamin D-25-hydroxylase (an enzyme that, in humans, is encoded by the *CYP2R1* gene-also known as cytochrome P450 2R1). The second metabolic step begins in the kidney, where 1-alpha-hydroxylase, an enzyme of the cytochrome P450 system, converts 25(OH)D to 1,25-(OH)2 D (or calcitriol, the biologically active form of vitamin D). Calcitriol then binds to vitamin D receptor (VDRs), which have been detected in almost every cell and tissue in the body [15,16]. By binding to these VDRs, vitamin D can control up to 200 genes connected with many health areas [14]. It is worth noting that the vitamin D bioactivity is a magnesium-dependent process because magnesium is an essential cofactor for vitamin D synthesis. As a result, activated vitamin D can increase intestinal absorption of magnesium. Moreover, there are evidences that magnesium supplementation can increase the effectiveness of vitamin D activity [17,18].

Vitamin D has numerous functions in the body, not only diminishing inflammation, but also influencing the immune systems, modulating cell growth, and controlling neuromuscular system [19,20]. In addition, vitamin D deficiency has been linked to many diseases and disorders, including infections, musculoskeletal disturbances, neuromuscular disorders, autoimmune diseases, diabetes and metabolic syndrome, lung and cardiovascular diseases, cognitive function and psychiatric disorders, and increased risk of some cancers [11,12,21]. Vitamin D deficiency is also connected with pain disorders, including fibromyalgia and headaches [12,22,23]. Despite these above-mentioned connections, causal relationships are yet to be established [22,24,25], and the underlying mechanism(s) by which vitamin D deficiency contributes to the above pathologies requires further investigation.

Another important issue is that a vitamin D status threshold level may be not sufficient enough to assess the individual requirements for vitamin D. Effectiveness of the molecular response to vitamin D, named as the vitamin D response index, differs between individuals. Thus the need for vitamin D supplementation may relay on the personal vitamin D response index rather than on the vitamin D status alone [26]. It should be also emphasized that the potency of vitamin D supplements from different manufacturers can vary widely [27].

### 1.2. The Role of Vitamin D in the Brain

As VDRs and 1-alpha-hydroxylase are present in many regions of the human brain (including the prefrontal cortex, thalamus, raphe, amygdala, cerebellum or, hippocampus), it is likely that vitamin D has specific functions in the central nervous system [15]. There is also evidence that vitamin D influence brain development: vitamin D deficient pups had larger brains, thinner neocortex and increased ventricular volume than controls in rodents [28]. Downregulation of genes controlling apoptosis is responsible for this changes in the brain, causing increased cellular proliferation [28]. Another important function of vitamin D is also regulation of the production of neurotrophic factors, (for example glial cell line-derived neurotrophic factor and nerve growth factor), thus it can act as a neuroprotective agent [16,28]. It is also a potent antioxidant, thus contributing to the vascular health of the brain [21]. Furthermore, vitamin D and its metabolites (as a steroid hormone) can influence many neurotransmitters, including dopamine, acetylcholine, and serotonin [23]. Indeed, some studies suggest vitamin D deficiency increases the risk of neurological diseases like stroke and dementia but also mood disorders [21]. Therefore, more and more data suggest they key role of vitamin D in maintaining brain health.

### 1.3. Vitamin D Deficiency and Pain

Several studies also indicate vitamin D deficiency can cause pain. In a meta-analysis of 81 observational studies, low vitamin D concentration was connected with arthritis, muscle pain, and chronic widespread pain [29]. However, the physiological mechanisms connecting vitamin D and pain is not fully known. The presence of VDR and vitamin D activating enzymes in the brain (especially the hypothalamus), and the influence of vitamin D on neurotransmitters, explains the connection between pain and vitamin D in fibromyalgia patients [15,23]. In addition, human and animal studies have shown insufficient vitamin D levels affect not only peripheral but also parasympathetic nerve function [23,30]. On the other hand, a Cochrane review published in 2015 showed vitamin D supplementation may not be better than placebo to control chronic pain in adults [31]. Therefore, there is some controversy surrounding the analgesic effects of vitamin D.

Kenis-Coskun et al. showed that although vitamin D replacement decrease pain and increase quality of life in patients with chronic widespread pain, it does not act via the spinal inhibitory circuit, ruling out any potential effect on the central pain sensitization mechanism [30]. In addition, other studies suggest the analgesic effect of vitamin D is more likely due to its anti-inflammatory action, rather than an anti-nociceptive effect [32]. Vitamin D appears to exert its anti-inflammatory effects by diminishing the release of pro-inflammatory cytokines and inhibiting T-cell responses [20]. For example, vitamin D shifts T-cell responses, increasing levels of T-helper (Th)-2 and regulatory T cells (Treg) instead of pro-inflammatory Th1 and Th17-cells [20,33]. In addition, in vitro studies show 25(OH)D inhibits the synthesis of prostaglandin E2 (PGE2) in fibroblasts [34]. There is also evidence that vitamin D supplementation stops musculoskeletal pain through diminished levels of inflammatory cytokines including PGE2 [35]. Thus, suppression of PGE2 is a credible explanation for the analgesic effect of vitamin D [35,36].

### 1.4. The Link between Vitamin D and Headache

In 2010, Prakash noticed that headache prevalence, including migraine, increase with increasing latitude [37]. Data also indicated that in autumn–winter frequency of headache attacks grow, while in summer, number of attacks decrease [37]. This pattern of headaches appears to match the serum vitamin D levels seasonal variations; however, the exact relationship between vitamin D deficiency and headache is somewhat enigmatic. Finding the link between vitamin D and migraine is made even more difficult due its complex pathophysiology, including metabolic, genetic, and hormonal elements that influence on the ability of the brain to process incoming sensory information [38]. Therefore, there are a number of ways by which vitamin D may influence primary headaches like migraines.

Inflammation plays a key role in migraine, whereby inflammatory substances produced by mast cells, mostly in the meninges, can activate the trigeminal nerve, a main structure involved in migraine headache [39,40]. Hence, the anti-inflammatory role of vitamin D may play an important part in migraine. In addition, in allergies, patients’ frequency of migraine attacks increases in certain seasons, which again suggests inflammation (and therefore vitamin D deficiency prompting excessive inflammation) may play an underlying role in primary headache. There is also an inverse association between the C-reactive protein (CRP, an inflammatory mediator) and vitamin D levels, and vitamin D supplementation can decrease inflammatory factors like CRP [33,41].

However, perhaps one of the most important mechanisms by which vitamin D deficiency could contribute to headache is through the possible sensitization of the second and third neurons, connected with stimulation of sensory receptors of the periosteal covering and central sensitization [30,42]. Another possible mechanism for headache associated with vitamin D deficiency is low serum levels of magnesium [43]. Magnesium plays an important role not only in neuromuscular conduction and nerve transmission, but also acts as a protective agent against excessive excitation causing neuronal cell death. The strong evidence exists regarding the close connection between magnesium and migraine, but also there are data suggesting a beneficial effect of magnesium for chronic pain conditions [44]. Intestinal absorption of magnesium depends on vitamin D, so diminished magnesium absorption due to vitamin D deficit may lead to TTH and migraine [17,18]. There is evidence that magnesium supplementation can be protective for migraine patients [8,9,10]. Thus, it is possible that the magnesium-associated benefit is partly mediated by vitamin D absorption and activation.

Vitamin D also reduces the production of nitric oxide (NO) by inhibiting the expression of NO synthase. NO is an important biological regulator that affects neurotransmission and vasodilation and is considered a key mediator in migraine [45]. During headaches attacks NO levels in jugular venous plasma increase; there are also evidence that NO synthase inhibitors are effective in treating migraine [46]. Moreover, in rodents, NO donors can enhance the release of the calcitonin gene-related protein (CGRP), which produce arterial vasodilatation and mast cell degranulation in the meninges. Moreover, NO controls the activity of spinal trigeminal neurons [46]. Therefore, it is possible that vitamin D diminish the frequency of migraine attacks by inhibiting NO synthase production.

Vitamin D also influence the release of dopamine and serotonin, which is known to be connected with the pathogenesis of migraine [38]. In particular, vitamin D can affect the synthesis of serotonin via tyrosine hydroxylase. So, in addition to its role in migraine pathogenesis, vitamin D deficiency may also cause depression, which often coexists with all types of headache [31].

More evidence of a potential connection between 25(OH)D and headache is the presence of VDRs, 1-alpha-hydroxylase, and the vitamin D binding protein (VDBP) in the brain, particularly in the hypothalamus [15,37,38,43]. In addition, the VDR gene, which is located on chromosome 12q, has several known polymorphisms that can produce different VDR proteins. The TaqI and FokI VDR polymorphisms have been shown to be associated with migraine without aura, and headache severity was found to be more significant in FokI heterozygote patients than in homozygote patients [47]. The explanation of this results is difficult, although may be connected with inflammation, because the relationship between polymorphisms of VDR, and susceptibility to several diseases associated with inflammation exist.

With regards to other types of primary headache, epidemiological studies show a strong relation between low serum vitamin D levels and chronic musculoskeletal pain. As chronic TTH patients can have generalized muscle pain, skull muscles tenderness, muscle atrophy and neck and other muscle weakness [48], it is speculated vitamin D deficiency is connected to TTH. In addition, CH shows a seasonal predilection, with nights attacks, suggesting involvement of the hypothalamus and vitamin D [3]. In particular, sunlight and vitamin D metabolism may be responsible for the diurnal and seasonal variation of CH. Indeed, evidence exists for diminished melatonin levels in CH patients, with decreased nocturnal serum melatonin levels during cluster periods [49].

**Figure 1:**
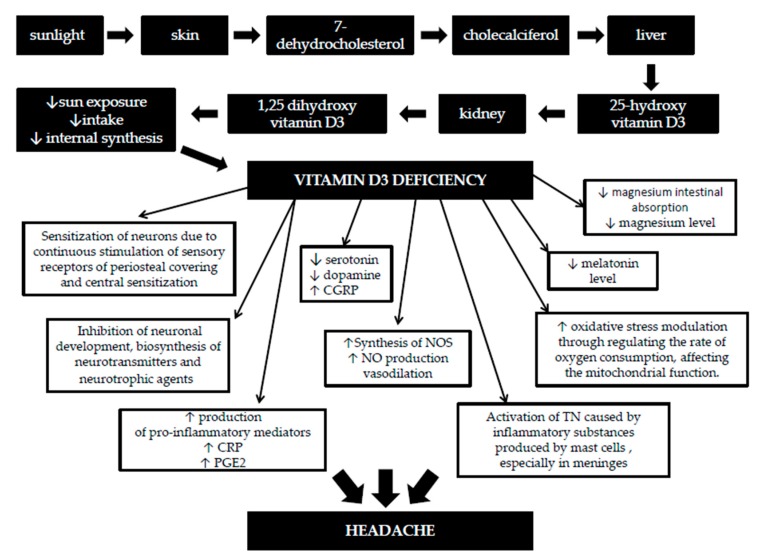
Potential role (s) that vitamin D deficiency can play in headache. Abbreviations: CGRP—calcitonin gene-related protein, NO—nitric oxide, NOS—nitric oxide synthase, CRP—C-reactive protein, PGE2—prostaglandin E2, TN—trigeminal nerve, ↓—a decrease, ↑—an increase.

Therefore, there are a number of possible connections between vitamin D deficiency and headache, as summarized in Figure 1. In this review article, we aimed to further examine the relationship between vitamin D and primary headache, and whether supplementation of vitamin D may be beneficial to certain patients.

## 2. Methods

This review included all articles regarding the connection between primary headache and vitamin D published up to October 2019. The list of studies was created by searching databases including: MEDLINE, EMBASE, PubMed, Google scholar, and the Cochrane library. Papers concerning the effects of vitamin D on headache were identified through a literature search. The following terminology and keywords were applied: “25-hydroxyvitamin D,” OR “vitamin D2,” OR “vitamin D3,” OR “ergosterol,” OR “cholecalciferol,” AND “headache”, OR “migraine,” OR “tension type headache,” OR “cluster headache,“ OR “trigeminal neuralgia,” OR “hemicranias,” OR “epidemiology,” OR “burden,” OR “treatment,” AND “immune function,” OR “inflammation,” OR “nociception,” OR “pain.” Only studies written in the English language were included. Articles including clinical trials, observational, cross-sectional and case-control studies and were included and reviewed.

## 3. Results and Discussion

Studies investigating vitamin D levels in association with primary headache are summarized in Table 1, and those studies that included the effect of vitamin D supplementation on headache are shown in Table 2. In most studies, vitamin D deficiency, insufficiency, and sufficiency were defined as <20, ≥20 and <30, and ≥30 ng/mL of 25(OH)D, respectively.

### 3.1. Vitamin D Deficiency and Tension-Type Headaches

Based on the studies published to date and summarized in Table 1, vitamin D deficiency is common among patients with TTH: low serum 25(OH)D levels were present in 67.2–73.0% of patients [22,57,60], and ranged from 13.5 to 16.9 ng/mL.

Prakash et al. examined 71 patients with chronic TTH, and found significantly lower mean 25(OH)D serum levels in subjects with musculoskeletal pain coexisting with headache comparing to those with headache only [22]. Similarly, patients having daily headaches had significantly lower serum vitamin D levels than those who had lower frequency of headaches [22]. The authors speculated that the coexistence of body pain and headache in a patient may be a symptom of a single disease, rather than two different disease or two separate mechanisms [22]. Moreover, if the mechanisms of musculoskeletal pain and of headache are the same and if osteomalacia of skull exist-headache may be a symptom of skull osteomalacia. Since headache can potentially coexist with pericranial tenderness, osteomalcia of the skull bone due to vitamin D deficiency could be a cause of TTH. The potential mechanism may be connected with swollen deposition of collagen rich osteoid on the periosteal surface of the skeleton which puts pressure on innervated periosteal covering. This continuous nociceptive inputs from the periphery because of swollen deposition of skull may sensitize the second and third order neurons and produce cephalic and extra cephalic pain. Another explanation is based on the study which demonstrated muscle hypersensitivity in rats receiving vitamin D-deficient diets. This muscles hypersensitivity together with and sensory hyperinnervation may exacerbate musculoskeletal pain [22]. Another study also showed 100 patients with chronic TTH had a significantly higher prevalence of musculoskeletal pain, muscle tenderness and weakness, and bone tenderness score compared to controls [60]. In addition, they showed vitamin D insufficiency was accompanied by an increased risk of chronic TTH [60]. Similarly, Hanci et al. found lower 25(OH)D levels in those with headache compared to controls, with not statistically significant difference [53].

### 3.2. The Role of Vitamin D in Migraine

Most studies revealed vitamin D deficiency or insufficiency in migraine patients, while some other studies showed normal vitamin D level (levels ranging from 12.40 to 38.08 ng/mL) [53,65]. Although some studies found no differences in vitamin D levels between the migraine and control groups [49,53,66], others found significant differences [54,55,65]. In particular, Togha et al. reported a higher serum vitamin D level (i.e., between 50 and 100 ng/mL) was associated with 80–83% lower odds of migraine headaches than those with serum levels below 20 ng/mL, after considering several confounding variables (including gender, age, and body mass index) [55]. They concluded that with each 5 ng/mL increase in serum 25(OH)D, there was 19–22% decreased odds of developing migraine [55]. Celikbilek et al. confirmed this observation: serum vitamin D and VDR levels were lower in migraineurs than in controls, with no significant differences in VDBP levels between the groups [65].

Meanwhile, a number of studies found no significant correlations between serum vitamin D levels and headache parameters, including aura, attack frequency, severity, and duration, and disease duration (Table 2). In particular, Montaghi and Zandifar showed no significant relationship between serum vitamin D and migraine severity [42,66]. In addition, some studies found no difference in serum 25(OH)D levels between episodic and chronic migraineurs [55,63]. Celibilek et al. also found no correlation between serum vitamin D, VDBP, and VDR levels and headache characteristics [65]. However, Rapsidaria et al. did discover a linear negative correlation between days with headache and serum vitamin D levels (Pearson’s correlation coefficient of 0.506; *p* < 0.001) [58], and Montaghi et al. observed a positive weak relationship between serum vitamin D level and headache diary result (*p* = 0.042, r = 0.19) [42]. Similarly, Song et al. found that a frequency of headache was related to vitamin D deficiency among migraineurs [56].

One study revealed a significantly higher incidence of aura, allodynia, phonophobia/photophobia, autonomic manifestations, and resistance to medications in migraineurs with vitamin D deficiency compared to those with normal vitamin D levels. There was also a statistically significant negative correlation between serum 25(OH)D level and the attack duration in hours (*p* < 0.001), frequency of the attacks/month (*p* < 0.001), migraine severity (MIGSEV) score (*p* = 0.001), and headache impact test (HIT)-6 score (*p* = 0.001) [54]. Likewise, a large population-based, cross-sectional study reported migraine severity was associated with vitamin D deficiency: the prevalence of moderate, major, and severe disability was higher among hospitalized migraineurs with vitamin D deficiency, and the mean length of stay and total cost of hospitalization increased (*p* < 0.001) [51]. Moreover, patients with vitamin D deficiency had a higher prevalence of migraine compared to those with hypocalcemia or without such deficiencies (3.0% vs. 1.5% vs. 1.6%, respectively; *p* < 0.0001) [51].

Furthermore, Buetner et al. noticed that in the presence of higher levels of vitamin D (57 nmol/l), statin use was associated with a significantly lower prevalence of severe headache or migraine, even after adjusting for multiple confounders (odds ratio [OR]: 0.48; 95% confidence interval [CI] 0.32–0.71, *p* < 0.001); however, no significant association between statin use and severe headache in those with low levels of vitamin D was observed [64].

### 3.3. Vitamin D in Cluster Headache

At present, only one study has examined serum vitamin D levels in patients with CH. Vitamin D deficiency was present in 92.8% of CH patients, with an average serum 25(OH)D concentration of 14.0 ± 3.9 ng/mL [49]. There was no difference in the serum 25(OH)D concentrations regarding gender, cluster and remission periods, first and recurrent attacks, or presence and absence of daily or seasonal periodicity. However, of the 14 patients with seasonal periodicity, those with a periodicity of winter to spring tended to have lower serum 25(OH)D concentrations than those with summer to autumn periodicity [49]. Therefore, more studies are required to confirm the relationship between vitamin D and CH, and determine whether supplementation may benefit these patients.

### 3.4. Association of Vitamin D with Trigeminal Neuralgia and Other Types of Primary Headache

Only one study examined the relationship between vitamin D and TN, and reported a significant decrease in vitamin D in patients compared to the control group [59]. There is currently no data regarding the association of vitamin D with other rare types of primary headache, including hemicrania, SUNCT, or hypnic headache.

### 3.5. Benefits of Vitamin D Supplementation in Headache

All except one study [24] showed a decrease in headache frequency after vitamin D supplementation (Table 2), and yet most studies revealed vitamin D supplementation had no impact on headache severity. A recent randomized, placebo-controlled parallel trial of vitamin D supplementation in patients with migraine showed a significant decrease in migraine frequency from baseline to week 24 compared with placebo (*p* < 0.001) [68]. The number of headache days reduced from 6.14 ± 3.60 to 3.28 ± 3.24 by the end of the trial in those taking vitamin D; however, there was no significant change in the migraine severity, pressure pain thresholds, or temporal summation [68]. Similarly, Buettner et al. proved that simvastatin plus vitamin D is beneficial for the prevention of headache in adults with episodic migraine, with the significant decrease in migraine frequency compared to placebo [69]. In the active treatment group, 8 patients (25%) experienced a 50% reduction in the number of migraine days at 12 weeks and 9 (29%) at 24 weeks post-randomization [69]. Simvastatin together with vitamin D was also associated with a significantly higher responder rate, and diminished migraine frequency, as well as doses of abortive migraine medications used [69].

Mottaghi et al. found no significant difference in the mean severity and duration of headache attacks due to vitamin D supplementation; however, migraine frequency was marginally reduced in the treatment group compared to controls (5.9 ± 7.0 vs. 7.0 ± 6.0; *p* = 0.06) [41]. In addition, the mean headache diary record was significantly lower in the treatment group compared to controls (85.0 ± 134.2 vs. 132.1 ± 147.1; *p* = 0.04) [41]. Similarly, a study among children with vitamin D insufficiency and deficiency showed migraine duration was significantly shorter (*p* < 0.001) after 6 months of vitamin D supplementation, and the migraine frequency, VAS scores, and PedMIDAS scores were all reduced [52]. In addition, Cayir et al. found the addition of vitamin D to current anti-migraine treatment (amitriptyline) reduced the number of migraine attacks in pediatric migraine patients compared with the group receiving amitriptyline treatment alone [72].

Conversely, Knutsen et al. concluded vitamin D supplementation had no significant effect on the occurrence, anatomical localization, and degree of pain parameters or headache [24]. This lack of effect may have been due to the low dose of daily vitamin D supplementation used in their study. In addition, the authors did not differentiate between the different types of headaches, so it is unclear what percentage of subjects were suffering migraines or other types of headache [24].

To date, no clinical trials have examined the influence of vitamin D supplementation specifically on TTH. In addition, only one poster has presented survey results of 110 CH sufferers using a daily dose of vitamin and mineral supplements, including 10,000 IU/day vitamin D3 and omega-3 fish oil as a CH preventative [71]. Overall, 80% of CH patients had significant reductions in frequency, duration, and severity of headache [71]. Therefore, although the initial results are promising, further studies on the benefits of vitamin D supplementation in the various types of headaches are warranted.

It is also worth noting that to date, vitamin D supplementation appears to be a safe form of treatment. Indeed, even at high doses of vitamin D (up to 10,000 IU/day), no major adverse events have been reported [71].

## 4. Conclusions

A large proportion of headache patients suffer from vitamin D deficiency. There is also some evidence indicating these patients have lower levels of vitamin D than healthy people. The strongest connection reported to date is between serum vitamin D levels and migraine headaches; more research is needed to establish connections between this vitamin and other types of headache. Although there is a link between vitamin D and headache frequency, a larger study should be performed to assess its connection with other headache characteristics. Based on our review of the current literature, there are not enough evidence to recommend vitamin D supplementation to all headache patients, but it may be beneficial in selected patients to reduce the frequency of headache, mainly in migraineurs, especially in those with vitamin D deficiency. Although, the optimal dose of vitamin D to be used in these patients requires determination.

## Figures and Tables

**Table 1:** Overview of studies investigating vitamin D serum levels in association with headache.

Author (Year)	Study Design	Study Group: Type of Headache (Number of Participants)	Study Population Age (Years)	Mean Serum Vitamin D Levels (ng/mL)	Results	Association
Gallelli (2019) [50]	Prospective, single-blind, single-center, control-group	Migraine (n = 95) Control (n = 120)	Range: 13–54	15.4	Serum vitamin D levels were lower than the normal range in patients with migraines and controls. Mean vitamin D values were significantly higher in the control group compared to the migraine group.	Associated
Patel (2019) [51]	Retrospective, cross-sectional	Migraine (n = 446, 446)	Range: 19–80	No data	Vitamin D deficiency elevated the odds of major/extreme loss of function. There was higher prevalence and higher odds of migraine among vitamin D deficient patients compared to those with hypocalcemia or no-deficiency.	Associated
Kılıç (2019) [52]	Retrospective study	Migraine (n = 92)	Mean: 12.6	No data	There was increased migraine frequency, duration, and PedMIDAS scores in those with vitamin D deficiency and insufficiency. Migraine frequency, duration, and PedMIDAS scores were significantly negatively related to serum vitamin D levels.	Associated
Hanci (2019) [53]	Retrospective, observational	Migraine (n = 165) TTH (n = 116) Control (n = 98)	Range: 5–17	Migraine: 12.4 ± 7.7 TTH: 13.5 ± 9.9 Control: 13.4 ± 8.8	No significant differences in mean vitamin D levels among the three groups	Not associated
Hussain (2019) [54]	Case-control	Migraine (n = 40) Control (n = 40)	32.18 28.8	Migraine: 32.11 Control: 41.86	Vitamin D levels were significantly lower in those with migraines compared to controls The incidence of aura, allodynia, phonophobia/photophobia, autonomic manifestations, and resistance to medications was increased vitamin D deficient patients with migraines than those with normal vitamin D levels. Serum vitamin D levels were significantly negatively related to the frequency, duration, and severity of migraine headache attacks	Associated
Togha (2018) [55]	Case-control	Migraine (n = 70) Control (n = 70)	Mean: 37	Migraine: 30 Control: 43	There were more subjects with vitamin D deficiency and insufficiency in the migraine group (53.7%) than the control group (26.1%). Serum vitamin D levels were significantly negatively related to migraine headaches Serum vitamin D levels in those with chronic migraine were not different to those in subjects with episodic migraine Serum vitamin D levels showed no correlation with headache parameters.	Associated
Song (2018) [56]	Retrospective, observational	Migraine (n = 157)	Mean: 37	15.9 ± 7.4	The majority (94.9%) of subjects with migraine had vitamin D insufficiency. Frequent monthly headache was 1.2 times more common in migraine patients with vitamin D deficiency than those without deficiency.	Associated
Donmez (2018) [57]	Retrospective, case-control	Migraine (n = 68) TTH (n = 79) Control (n = 69)	Mean: 12.2	Migraine: 17.3 TTH: 16.9 Control: 25.8	Serum vitamin D levels were significantly lower in the migraine and TTH groups compared with the control group.	Associated
Sohn (2018) [49]	Case-control	CH (n = 28) Migraine (n = 36) Control (n = 36)	CH: 38.2 Migraine: 35.1 Control: 35.4	CH: 14.0 ± 3.9 Migraine: 14.7 ± 5.9 Control: 14.6 ± 7.4	The majority (92.8%) of those with CH had vitamin D deficiency. There was no significant difference in vitamin D levels among patients with CH, migraine, or controls. Patients with a headache periodicity during winter to spring showed a trend of lower serum vitamin D levels than those with periodicity during summer to autumn.	Not associated
Rapisarda (2018) [58]	Case-control	CM (n = 100) EM (n = 34) Control (n = 38)	CM/EM: 41.4 Control: 47.6	CM: 12.7 EM: 17.2 Control: 23.0	Vitamin D deficiency was severe among headache patients (especially in those with CM) compared to healthy subjects. Vitamin D levels were negatively correlated with the number of days of headache (Pearson’s correlation coefficient: 0.506)	Associated
Farajzadeh (2018) [59]	Case-control	TN (n = 13) Control (n = 13)	Mean: 53.3	TN: 22.61 Control: 39.80	Vitamin D levels were significantly decreased in patients with TN (before and after microvascular decompression) compared to the control group.	Associated
Prakash (2017) [60]	Case-control	Chronic TTH (n = 100) Control (n = 100)	Chronic TTH: 35.63 Control: 36.86	Chronic TTH: 14.7 Control: 27.4	Serum vitamin D levels were significantly lower in Chronic TTH patients than in controls. Vitamin D deficiency was more prevalent in patients with Chronic TTH (71%) than controls (25%). Chronic TTH patients with vitamin D deficiency had a higher prevalence of musculoskeletal pain, muscle weakness, muscle and bone tenderness score, associated fatigue, and a more prolonged course. Serum vitamin D levels were positively correlated with the total muscle tenderness score.	Associated
Virtanen (2017) [61]	Cross-sectional	Self-reported frequent headache (n = 250)	Range: 42–60	38.3 nmol/L	Serum vitamin D levels were lower in subjects with frequent headaches than other participants. Serum vitamin D levels were inversely associated with frequent headaches.	Associated
Tozzi (2016) [62]	Cross-sectional	MWoA (n = 91) MWA (n = 32) TTH (n = 36)	Range: 5–18	No data	Serum vitamin D levels were lower in children with MwoA than those with MWA and THH, albeit not significantly (*p* = 0.07).	Not associated
Iannacchero (2015) [63]	Observational	Migraine (n = 22)	Mean: 45.41	13.05 ± 5.70	Vitamin D levels were similar among those with CM than those with EM Vitamin D levels were not significantly correlated with headache frequency.	Not associated
Buettner (2015) [64]	Cross-sectional	5938 participants from the National Health and Nutrition Examination	No data	No data	People with serum vitamin D levels >57 nmol/l and use a statin had a lower prevalence of severe headache or migraine.	Associated
Prakash (2013) [22]	Observational	Chronic TTH (n = 71)	Mean: 38	No data	Serum vitamin D levels were significantly associated with headache, musculoskeletal pain, and osteomalacia. Mean vitamin D levels were significantly lower in subjects suffering from daily headache compared to those with intermittent headaches.	Associated
Celikbilek (2014) [65]	Cross-sectional, prospective	Migraine (n = 52) Control (n = 49)	Migraine: 35.88 Control: 34.24	Migraine: 38.08 Control: 48.03	Serum vitamin D and VDR levels were significantly lower in migraine patients than controls. Serum VDBP levels were similar between the two groups. Serum vitamin D, VDBP, and VDR levels showed no correlated with headache characteristics.	Associated
Zandifar (2014) [66]	Case-control	Migraine (n = 105) Control (n = 110)	Migraine: 33.59 Control: 32.46	Migraine: 13.55 ± 0.91 Control: 13.19 ± 1.19	There was no significant difference in vitamin D levels among between case controls. Severity of headache was not related to vitamin D levels.	Not associated
Mottaghi (2013) [42]	Cross-sectional	Migraine (n = 76)	Mean: 33.1	23.3 ± 1.8	Serum vitamin D were weakly positively associated with headache diary result but not related to migraine severity High serum levels of 25-OH-D3 were related to a higher headache diary result.	Associated
Kjaergaard (2012) [67]	Cross-sectional	11,614 participants of the sixth survey of the Tromsø study in 2007–2008	Range: 55–58	No data	Serum vitamin D levels were inversely associated with non-migraine headache but there was no significant association between migraine and serum vitamin D.	Associated
Knutsen (2014) [24]	Cross-sectional	Headache (n = 63)	No data	No data	Mean serum vitamin D levels in patients with headaches were lower than in those suffering from musculoskeletal pain or fatigue. Headache was inversely associated with hypovitaminosis D.	Associated

Abbreviations: CH–cluster headache; CM–chronic migraine; EM–episodic migraine; MWA–migraine with aura; MWoA–migraine without aura; PedMIDAS–Pediatric Migraine Disability Assessment; TN–trigeminal neuralgia; TTH–tension type headache; VDBP –vitamin D binding protein; VDR–vitamin D receptor.

**Table 2:** Overview of studies evaluating the effect of vitamin D supplementation on headache parameters.

Author (Year)	Study Design	Study Group: Type of Headache (Number of Participants)	Study Population Age (Years)	Supplementation Period	Vitamin D Dosage	Mean Serum 25(OH)D Levels (ng/mL) and/or 1,25(OH)2D (pg/mL) Before Treatment	Mean Serum 25(OH)D Levels (ng/mL) and/or 1,25(OH)2D (pg/mL) After the Treatment	Results
Kılıç (2019) [52]	Prospective	Migraine (n = 42)	Mean: 14	8 months	2000 IU/day for 2 months, then 600–1000 IU/day of maintenance therapy for the next 6 months	25(OH)D 9.4 (4.2–20)	25(OH)D 34.6 (16.3–45)	Decreased migraine duration, frequency, VAS scores, and PedMIDAS scores compared with baseline values. No effect on headache severity.
Gazerani (2019) [68]	Randomized, double-blind, placebo-controlled, parallel	Migraine (n = 48)	Mean: 45.5	196 days	100 μg/day	25(OH)D 87.43 ± 32.00 1,25(OH)2D 43.55 ± 10.57	25(OH)D Increased significantly 1, 25(OH)2D No significant change Values not provided	Decreased migraine frequency, but no effect on severity, pressure pain thresholds, or temporal summation.
Buettner (2016) [69]	Randomized, placebo-controlled	Episodic migraine (n = 57)	Mean: 40	24 weeks	1000 IU twice per day (+ simvastatin 20 mg/twice per day)	25(OH)D 30 (18 to 41) median (IQR)	25(OH)D 38 (34 to 45 median (IQR	Decreased number of migraine days.
Yilmaz (2016) [70]	Pre-post	Headache (n = 29)	Mean: 36.9	3 months	50,000 IU/weekly + calcium of 1000 mg/day	25(OH)D 10.6 ± 5.1	25(OH)D 46.5 ± 24.0	Decreased headache severity and frequency.
Mottaghi (2015) [41]	Randomized, double-blind placebo-controlled	Migraine (n = 65)	Range: 10–65	10 weeks	50,000 IU/week	25(OH)D 16 ± 5.4	25(OH)D No data	Decreased headache frequency and mean headache diary results, but no effect on the severity and duration of headache.
Knutsen (2014) [24]	Randomized double-blinded placebo-controlled parallel-group	Headache (n = 157)	Range: 35–40	16 weeks	Group 1 25 μg/day Group 2 10 μg/day	25(OH)D Group 1 27 Group 2 25	25(OH)D Group 1 increased by 25 Group 2 increased by 16	No effect on the occurrence, anatomical localization, and degree of pain parameters or headache frequency.
Batcheller (2014) [71]	Prospective	Cluster headache (n = 110)	No data	30 days	10,000 IU/day	25(OH)D 23.4	25(OH)D 76	Decreased frequency, severity, and duration of headache in 80% of patients.
Cayir (2014) [72]	Prospective	Migraine (n = 53)	Range: 8–16	6 months	Group 1: amitriptyline alone Group 2: 400 IU/day + amitriptyline Group 3: 800 IU/day + amitriptyline Group 4: 5000 IU/day + amitriptyline	Group 1 32.4 ± 2 Group 2 28.1 ± 1.8 Group 3 17.2 ± 0.3 Group 4: 10.9 ± 0.6	Group 1 33.7 ± 1.8 Group 2 34.1 ± 1.6 Group 3 25.6 ± 1.1 Group 4: 22.3 ± 1.9	Decreased headache attack frequency in groups 2, 3, and 4

Abbreviations: PedMIDAS–Pediatric Migraine Disability Assessment; VAS–visual assessment scale.

## References

[1] Headache Classification Committee of the International Headache Society (IHS) (2018). The International Classification of Headache Disorders, 3rd edition. Cephalalgia.

[2] Collaborators G.H. (2018). Global, regional, and national burden of migraine and tension-type headache, 1990–2016: A systematic analysis for the Global Burden of Disease Study 2016. Lancet Neurol..

[3] Wei D.Y., Yuan Ong J.J., Goadsby P.J. (2018). Cluster Headache: Epidemiology, Pathophysiology, Clinical Features, and Diagnosis. Ann. Indian Acad. Neurol..

[4] Zakrzewska J.M., Linskey M.E. (2014). Trigeminal neuralgia. BMJ Clin. Evid..

[5] Tölle T., Dukes E., Sadosky A. (2006). Patient burden of trigeminal neuralgia: Results from a cross-sectional survey of health state impairment and treatment patterns in six European countries. Pain Pract..

[6] Stovner L.J., Zwart J.A., Hagen K., Terwindt G.M., Pascual J. (2006). Epidemiology of headache in Europe. Eur. J. Neurol..

[7] Abu Bakar N., Tanprawate S., Lambru G., Torkamani M., Jahanshahi M., Matharu M. (2016). Quality of life in primary headache disorders: A review. Cephalalgia.

[8] Nattagh-Eshtivani E., Sani M.A., Dahri M., Ghalichi F., Ghavami A., Arjang P., Tarighat-Esfanjani A. (2018). The role of nutrients in the pathogenesis and treatment of migraine headaches: Review. Biomed. Pharmacother..

[9] Martin V.T., Vij B. (2016). Diet and Headache: Part 2. Headache.

[10] Orr S.L. (2018). The Evidence for the Role of Nutraceuticals in the Management of Pediatric Migraine: A Review. Curr. Pain Headache Rep..

[11] Holick M.F. (2017). The vitamin D deficiency pandemic: Approaches for diagnosis, treatment and prevention. Rev. Endocr. Metab. Disord..

[12] Autier P., Boniol M., Pizot C., Mullie P. (2014). Vitamin D status and ill health: A systematic review. Lancet Diabetes Endocrinol..

[13] Lee J.H., O’Keefe J.H., Bell D., Hensrud D.D., Holick M.F. (2008). Vitamin D deficiency an important, common, and easily treatable cardiovascular risk factor?. J. Am. Coll. Cardiol..

[14] Holick M.F., Binkley N.C., Bischoff-Ferrari H.A., Gordon C.M., Hanley D.A., Heaney R.P., Murad M.H., Weaver C.M., Society E. (2011). Evaluation, treatment, and prevention of vitamin D deficiency: An Endocrine Society clinical practice guideline. J. Clin. Endocrinol. Metab..

[15] Eyles D.W., Smith S., Kinobe R., Hewison M., McGrath J.J. (2005). Distribution of the vitamin D receptor and 1 alpha-hydroxylase in human brain. J. Chem. Neuroanat..

[16] Trochoutsou A.I., Kloukina V., Samitas K., Xanthou G. (2015). Vitamin-D in the Immune System: Genomic and Non-Genomic Actions. Mini Rev. Med. Chem..

[17] Razzaque M.S. (2018). Magnesium: Are We Consuming Enough?. Nutrients.

[18] Uwitonze A.M., Razzaque M.S. (2018). Role of Magnesium in Vitamin D Activation and Function. J. Am. Osteopath. Assoc..

[19] Martin K.R., Reid D.M. (2017). Is there role for vitamin D in the treatment of chronic pain?. Ther. Adv. Musculoskelet. Dis..

[20] Hewison M. (2012). Vitamin D and immune function: An overview. Proc. Nutr. Soc..

[21] Holick M.F. (2015). Vitamin D and brain health: The need for vitamin D supplementation and sensible sun exposure. J. Intern. Med..

[22] Prakash S., Kumar M., Belani P., Susvirkar A., Ahuja S. (2013). Interrelationships between chronic tension-type headache, musculoskeletal pain, and vitamin D deficiency: Is osteomalacia responsible for both headache and musculoskeletal pain?. Ann. Indian Acad. Neurol..

[23] Karras S., Rapti E., Matsoukas S., Kotsa K. (2016). Vitamin D in Fibromyalgia: A Causative or Confounding Biological Interplay?. Nutrients.

[24] Knutsen K.V., Madar A.A., Brekke M., Meyer H.E., Natvig B., Mdala I., Lagerløv P. (2014). Effect of vitamin D on musculoskeletal pain and headache: A randomized, double-blind, placebo-controlled trial among adult ethnic minorities in Norway. Pain.

[25] Yang Y., Zhang H.L., Wu J. (2010). Is headache related with vitamin D insufficiency?. J. Headache Pain.

[26] Carlberg C., Haq A. (2018). The concept of the personal vitamin D response index. J. Steroid Biochem. Mol. Biol..

[27] LeBlanc E.S., Perrin N., Johnson J.D., Ballatore A., Hillier T. (2013). Over-the-counter and compounded vitamin D: Is potency what we expect?. JAMA Intern. Med..

[28] Groves N.J., McGrath J.J., Burne T.H. (2014). Vitamin D as a neurosteroid affecting the developing and adult brain. Annu. Rev. Nutr..

[29] Wu Z., Malihi Z., Stewart A.W., Lawes C.M., Scragg R. (2018). The association between vitamin D concentration and pain: A systematic review and meta-analysis. Public Health Nutr..

[30] Kenis-Coskun O., Giray E., Gunduz O.H., Akyuz G. (2019). The effect of Vitamin D replacement on spinal inhibitory pathways in women with chronic widespread pain. J. Steroid Biochem. Mol. Biol..

[31] Straube S., Derry S., Straube C., Moore R.A. (2015). Vitamin D for the treatment of chronic painful conditions in adults. Cochrane Database Syst. Rev..

[32] Gaikwad M., Vanlint S., Mittinity M., Moseley G.L., Stocks N. (2017). Does vitamin D supplementation alleviate chronic nonspecific musculoskeletal pain? A systematic review and meta-analysis. Clin. Rheumatol..

[33] Hewison M. (2011). Antibacterial effects of vitamin D. Nat. Rev. Endocrinol..

[34] Liu X., Nelson A., Wang X., Farid M., Gunji Y., Ikari J., Iwasawa S., Basma H., Feghali-Bostwick C., Rennard S.I. (2014). Vitamin D modulates prostaglandin E2 synthesis and degradation in human lung fibroblasts. Am. J. Respir. Cell Mol. Biol..

[35] Helde-Frankling M., Björkhem-Bergman L. (2017). Vitamin D in Pain Management. Int. J. Mol. Sci..

[36] Gendelman O., Itzhaki D., Makarov S., Bennun M., Amital H. (2015). A randomized double-blind placebo-controlled study adding high dose vitamin D to analgesic regimens in patients with musculoskeletal pain. Lupus.

[37] Prakash S., Mehta N.C., Dabhi A.S., Lakhani O., Khilari M., Shah N.D. (2010). The prevalence of headache may be related with the latitude: A possible role of Vitamin D insufficiency?. J. Headache Pain.

[38] Goadsby P.J., Holland P.R. (2019). An Update: Pathophysiology of Migraine. Neurol. Clin..

[39] Levy D., Burstein R., Kainz V., Jakubowski M., Strassman A.M. (2007). Mast cell degranulation activates a pain pathway underlying migraine headache. Pain.

[40] Burstein R., Noseda R., Borsook D. (2015). Migraine: Multiple processes, complex pathophysiology. J. Neurosci..

[41] Mottaghi T., Askari G., Khorvash F., Maracy M.R. (2015). Effect of Vitamin D supplementation on symptoms and C-reactive protein in migraine patients. J. Res. Med. Sci..

[42] Mottaghi T., Khorvash F., Askari G., Maracy M.R., Ghiasvand R., Maghsoudi Z., Iraj B. (2013). The relationship between serum levels of vitamin D and migraine. J. Res. Med. Sci..

[43] Prakash S., Shah N.D. (2009). Chronic tension-type headache with vitamin D deficiency: Casual or causal association?. Headache.

[44] Kirkland A.E., Sarlo G.L., Holton K.F. (2018). The Role of Magnesium in Neurological Disorders. Nutrients.

[45] Garcion E., Sindji L., Nataf S., Brachet P., Darcy F., Montero-Menei C.N. (2003). Treatment of experimental autoimmune encephalomyelitis in rat by 1,25-dihydroxyvitamin D3 leads to early effects within the central nervous system. Acta Neuropathol..

[46] Messlinger K., Lennerz J.K., Eberhardt M., Fischer M.J. (2012). CGRP and NO in the trigeminal system: Mechanisms and role in headache generation. Headache.

[47] Motaghi M., Haghjooy Javanmard S., Haghdoost F., Tajadini M., Saadatnia M., Rafiee L., Zandifar A. (2013). Relationship between vitamin D receptor gene polymorphisms and migraine without aura in an Iranian population. Biomed. Res. Int..

[48] Fernández-de-Las-Peñas C., Cuadrado M.L., Arendt-Nielsen L., Ge H.Y., Pareja J.A. (2007). Increased pericranial tenderness, decreased pressure pain threshold, and headache clinical parameters in chronic tension-type headache patients. Clin. J. Pain.

[49] Sohn J.H., Chu M.K., Park K.Y., Ahn H.Y., Cho S.J. (2018). Vitamin D deficiency in patients with cluster headache: A preliminary study. J. Headache Pain.

[50] Gallelli L., Michniewicz A., Cione E., Squillace A., Colosimo M., Pelaia C., Fazio A., Zampogna S., Peltrone F., Iannacchero R. (2019). 25-Hydroxy Vitamin D Detection Using Different Analytic Methods in Patients with Migraine. J. Clin. Med..

[51] Patel U., Kodumuri N., Malik P., Kapoor A., Malhi P., Patel K., Saiyed S., Lavado L., Kapoor V. (2019). Hypocalcemia and Vitamin D Deficiency amongst Migraine Patients: A Nationwide Retrospective Study. Medicina (Kaunas).

[52] Kılıç B., Kılıç M. (2019). Evaluation of Vitamin D Levels and Response to Therapy of Childhood Migraine. Medicina (Kaunas).

[53] Hancı F., Kabakuş N., Türay S., Bala K.A., Dilek M. (2019). The role of obesity and vitamin D deficiency in primary headaches in childhood. Acta Neurol. Belg..

[54] Hussein M., Fathy W., Elkareem R.M.A. (2019). The potential role of serum vitamin D level in migraine headache: A case–control study. Journal of Pain Research.

[55] Togha M., Razeghi Jahromi S., Ghorbani Z., Martami F., Seifishahpar M. (2018). Serum Vitamin D Status in a Group of Migraine Patients Compared With Healthy Controls: A Case-Control Study. Headache.

[56] Song T.J., Chu M.K., Sohn J.H., Ahn H.Y., Lee S.H., Cho S.J. (2018). Effect of Vitamin D Deficiency on the Frequency of Headaches in Migraine. J. Clin. Neurol..

[57] Donmez A., Orun E., Sonmez F.M. (2018). Vitamin D status in children with headache: A case-control study. Clin. Nutr. ESPEN.

[58] Rapisarda L., Mazza M.R., Tosto F., Gambardella A., Bono F., Sarica A. (2018). Relationship between severity of migraine and vitamin D deficiency: A case-control study. Neurol. Sci..

[59] Farajzadeh A., Bathaie S.Z., Arabkheradmand J., Ghodsi S.M., Faghihzadeh S. (2018). Different Pain States of Trigeminal Neuralgia Make Significant Changes in the Plasma Proteome and Some Biochemical Parameters: A Preliminary Cohort Study. J. Mol. Neurosci..

[60] Prakash S., Rathore C., Makwana P., Dave A., Joshi H., Parekh H. (2017). Vitamin D Deficiency in Patients With Chronic Tension-Type Headache: A Case-Control Study. Headache.

[61] Virtanen J.K., Giniatullin R., Mäntyselkä P., Voutilainen S., Nurmi T., Mursu J., Kauhanen J., Tuomainen T.P. (2017). Low serum 25-hydroxyvitamin D is associated with higher risk of frequent headache in middle-aged and older men. Sci. Rep..

[62] Tozzi E., Boncristiano A., Antenucci A., Di Loreto S., Farello G. (2015). P013. 25(OH)D Level and headache in children sample. J. Headache Pain.

[63] Iannacchero R., Costa A., Squillace A., Gallelli L., Cannistrà U., De Sarro G. (2015). P060. Vitamin D deficiency in episodic migraine, chronic migraine and medication-overuse headache patients. J. Headache Pain.

[64] Buettner C., Burstein R. (2015). Association of statin use and risk for severe headache or migraine by serum vitamin D status: A cross-sectional population-based study. Cephalalgia.

[65] Celikbilek A., Gocmen A.Y., Zararsiz G., Tanik N., Ak H., Borekci E., Delibas N. (2014). Serum levels of vitamin D, vitamin D-binding protein and vitamin D receptor in migraine patients from central Anatolia region. Int. J. Clin. Pract..

[66] Zandifar A., Masjedi S.S., Banihashemi M., Asgari F., Manouchehri N., Ebrahimi H., Haghdoost F., Saadatnia M. (2014). Vitamin D status in migraine patients: A case-control study. Biomed. Res. Int..

[67] Kjaergaard M., Eggen A.E., Mathiesen E.B., Jorde R. (2012). Association between headache and serum 25-hydroxyvitamin D: The Tromsø Study: Tromsø 6. Headache.

[68] Gazerani P., Fuglsang R., Pedersen J.G., Sørensen J., Kjeldsen J.L., Yassin H., Nedergaard B.S. (2019). A randomized, double-blinded, placebo-controlled, parallel trial of vitamin D. Curr. Med. Res. Opin..

[69] Buettner C., Nir R.R., Bertisch S.M., Bernstein C., Schain A., Mittleman M.A., Burstein R. (2015). Simvastatin and vitamin D for migraine prevention: A randomized, controlled trial. Ann. Neurol..

[70] Yilmaz R., Salli A., Cingoz H.T., Kucuksen S., Ugurlu H. (2016). Efficacy of vitamin D replacement therapy on patients with chronic nonspecific widespread musculoskeletal pain with vitamin D deficiency. Int. J. Rheum. Dis..

[71] Batcheller P. (2014). A Survey of Cluster Headache (CH) Sufferers Using Vitamin D3 as a CH Preventative (P1. 256).

[72] Cayir A., Turan M.I., Tan H. (2014). Effect of vitamin D therapy in addition to amitriptyline on migraine attacks in pediatric patients. Braz. J. Med. Biol. Res..

